# Epstein–Barr virus and cytomegalovirus coinfection in Egyptian COVID-19 patients

**DOI:** 10.1186/s43066-023-00262-y

**Published:** 2023-05-23

**Authors:** Eman F Barakat, Ahmed F Sherief, Nesma G Elsheikh, Mai Mohi M. El-Metwaly Khalifa

**Affiliations:** 1 Tropical Medicine Department, Faculty of Medicine, Ain Shams University, Cairo, Egypt; 2 Geriatric and Gerontology Medicine, Faculty of Medicine, Ain Shams University, Cairo, Egypt; 3 Tropical Medicine Department, Damietta Fever Hospital, Damietta, Egypt

**Keywords:** COVID-19, EBV, CMV, SARS-CoV-2

## Abstract

**Background:**

Reactivation of herpesviruses such as Epstein–Barr virus (EBV) and cytomegalovirus (CMV) in COVID-19 patients reported in many studies in different countries during the pandemic. We aimed to measure prevalence of this coinfection in Egyptian COVID-19 patients with elevated liver enzymes and its relation to the severity and the outcome of COVID-19 infection in those patients.

**Methods:**

A cross-sectional study was carried out on 110 COVID-19 patients with elevated liver enzymes regardless the severity of COVID-19 disease. All patients were subjected to medical history, clinical examination, laboratory investigations, high-resolution computed tomography chest (HRCT chest). Epstein–Barr virus (EBV) and Human cytomegalovirus (HCMV) were determined by VCA IgM and CMV IgM respectively by enzyme-linked immunosorbent assay (ELISA).

**Results:**

Of the included 110 patients with COVID-19 illness, 5 (4.5%) were Epstein–Barr virus seropositive and 5 (4.5%) were human cytomegalovirus seropositive. Regarding the symptoms, the incidence of fever in the EBV and CMV seropositive group was apparently higher than that in the EBV and CMV seronegative group. In lab tests, the platelets and albumin of EBV and CMV seropositive group decreased more significantly than EBV and HCMV seronegative group, and serum ferritin, D-dimer, and C-reactive protein show higher values in seropositive group than in seronegative group but not statistically significant. Seropositive group had received higher doses of steroids than seronegative group. The median of hospital stay in seropositive group was (15 days) nearly double that of seronegative group with statistically significant difference between both groups.

**Conclusion:**

Coinfection of EBV and CMV in COVID-19 Egyptian has no effect on the disease severity or the clinical outcome of the disease. But those patients had higher hospital stay duration.

## Introduction

The novel coronavirus first detected in Wuhan, China, in December 2019, named severe acute respiratory syndrome-coronavirus-2 (SARS-COV-2), causes coronavirus disease 2019 (COVID-19) [1]. COVID-19 is primarily transmitted through contact with droplets which contain viral particles [2].

A strong immune response is triggered in the lungs by the rapid replication of SARS-CoV-2. Cytokine storm syndrome gives rise to acute respiratory distress syndrome and respiratory failure, that is considered the main cause of death in COVID-19 patients [3, 4]. In some COVID-19 cases, multiple organ failure has also been reported [5, 6]. Increasing levels of ALT, AST, and bilirubin in SARS-CoV-2 infection, indicating injury to the hepatic tissue [3–7]. Some of these liver serum enzymes indicating hepatic damage, albumin level was significantly decreased, indicating the severity of the infection [8].

It was reported that there is suppression in COVID-19 patients’ immunity in the early stage of the disease particularly T cell-mediated immunity [9, 10]. Furthermore, drugs used in COVID‐19 treatment may also act a major role in altering immune responses by means of regulating intracellular signaling pathways, hence prompting the reactivation process of EBV. High dose of corticosteroids, which is used in COVID-19 treatment, has been stated as a risk factor for herpes virus reactivation, particularly EBV and CMV [11, 12].

## Methods

### Study design and participants

This was a cross-sectional study to measure prevalence of Epstein–Barr virus and cytomegalovirus in elevated liver enzymes patients infected with COVID-19. And to evaluate the relation between the severity and COVID-19 infection outcome in patients coinfected with CMV and EBV. One hundred and ten COVID-19 patients with elevated liver enzymes presented to Ain Shams University Hospitals and Damietta Fever Hospital. All adult patients (the mean age of patients is 55), with variety of spectrum of COVID‐19 illness, ranges from mild up to critical case, confirmed by qualitative real-time RT-PCR assay of nasopharyngeal swabs or by (SARS-COV2 antigen-RDT) and radiological criteria for COVID-19 infection [13], were included in the study. The spectrum of COVID-19 disease severity is classified, based on the severity of symptoms into four levels: mild, moderate, severe, and critical. Mild cases only have mild symptoms without radiographic features. Moderate cases present with fever, respiratory symptoms, and radiographic features. Severe cases develop one of three criteria: (a) dyspnea, RR more than 30 times/min, (b) oxygen saturation less than 93% in ambient air, and (c) PaO_2_/FiO_2_ less than 300 mmHg. Critical cases develop one of three criteria: (a) respiratory failure, (b) septic shock, and (c) multiple organ failure.

Patients with pre-existing liver disease, such as:viral hepatitis (A, B, C), decompensated liver disease, and hepatocellular carcinoma, were excluded from the study.

### Study tools

Serum samples (2 ml) studied for EBV (viral capsid antigen) VCA IgM and CMV IgM determined by enzyme-linked immunosorbent assay (ELISA). All patients were tested for CBC, CRP, ALT, AST, D-dimer, and serum ferritin.

### Statistical analysis

- Data were tabulated and statistically analyzed using SPSS, version 20 (SPSS Inc., Chicago, IL).
- Quantitative data were described as mean and standard deviation (minimum–maximum) and median and interquartile range.
- Mann-Whitney *U* test was used for comparing quantitative variables between groups.
- Qualitative data were expressed as frequencies (*n*) and percentage (%).
- Fisher exact test was used to test the association between qualitative variables.
- *P* value ≤ 0.05 was considered statistically significant.

## Results

Baseline demographic data of patients included in this study, are listed in the Table 1. The mean age of patients was 55 ± 14.4 (range, 23–87 years) and 60.0% were less than or equal 60 years, with 50.9% were males. 94.5% of patients have no special habits, 75.5% were from Damietta, 57.6% (*n* = 49) were retired or not working.

**Table 1:** Sociodemographic characteristics distribution among the studied patients (*n* = 110)

	***N***	**%**
Age (years) *[mean* ± *SD (min–max)]*	55 ± 14.4 (23–87)	
Age		
Less than or equal 60 years	66	60.0%
More than 60 years	44	40.0%
Gender		
Male	56	50.9%
Female	54	49.1%
Special habits		
No special habits	104	94.5%
Smoking	5	4.5%
Smoking, alcoholic, and addiction	1	0.9%
CT chest findings		
Bilateral subpleural GGOs patches	98	89.1%
Unilateral subpleural GGOs patches	3	2.7%
Free C.T chest	1	0.9%
Others	14	12.7%

Others include consolidation, broncho-vascular marking, and atelectatic bands

*GGOs* ground glass opacities

CT chest findings among COVID-19 participants in the current study are listed in the Table 1. Shows that the most common finding was bilateral ground glass opacity (GGOs) (89.1%) while the other finding was unilateral subpleural GGOs patches as well as free CT chest.

There were 10 (9.1%) patients with either CMV or EBV seropositive tests group among the studied group of COVID-19 patients. Among those 10 patients, 5 were CMV and the other were EBV seropositive.

As regards comparison of sociodemographic data between COVID-19 patients with and without CMV or EBV coinfection, Table 2 shows that there were (60.0%) of seropositive group for EBV-VCA IgM and CMV IgM less than or equal 60 years old and it was not statistically significant difference. As regards gender, males (60%) were more than females with no statistically significant difference. As regards special habits of medical importance, 90.0% of patients have no special habits, and there was no statistically significant difference between smokers and non-smokers.

**Table 2:** Comparison of sociodemographic data and comorbidities between COVID-19 patients with and without CMV or EBV coinfection (*n* = 110)

	Either CMV or EBV				*P*
	Negative (*n* = 100)		Positive (*n* = 10)		
	*N*	%	*N*	%	
Age					
Less than or equal 60 years	60	60.0%	6	60.0%	1.000
More than 60 years	40	40.0%	4	40.0%	
Gender					
Male	50	50.0%	6	60.0%	.742
Female	50	50.0%	4	40.0%	
Special habits					
No special habits	95	95.0%	9	90.0%	.151
Smoking	5	5.0%	0	0.0%	
Smoking, alcoholic, and addiction	0	0.0%	1	10.0%	
No comorbidities					
No	54	54.0%	7	70.0%	.507
Yes	46	46.0%	3	30.0%	
DM					
No	62	62.0%	5	50.0%	.509
Yes	38	38.0%	5	50.0%	
HTN					
No	64	64.0%	5	50.0%	.496
Yes	36	36.0%	5	50.0%	
IHD					
No	92	92.0%	9	90.0%	.590
Yes	8	8.0%	1	10.0%	
BA and COPD					
No	96	96.0%	10	100.0%	1.000
Yes	4	4.0%	0	0.0%	
Cardiomyopathy and HF					
No	98	98.0%	9	90.0%	.251
Yes	2	2.0%	1	10.0%	
kidney disease					
No	99	99.0%	8	80.0%	.021*
Yes	1	1.0%	2	20.0%	
Others					
No	97	97.0%	9	90.0%	.321
Yes	3	3.0%	1	10.0%	
No comorbidities					
No	54	54.0%	7	70.0%	.507
Yes	3	3.0%	1	10.0%	

*DM* diabetes mellitus, *HTN* hypertension, *IHD* ischemic heart disease, *BA* bronchial asthma, *COPD* chronic obstructive pulmonary disease, *HF* heart failure

Fisher’s exact test was used; (*) *P* value (≤ 0.05) is considered statistically significant

Othersincludes old poliomyelitis, myasthenia gravis, leukemia, cancer prostate, and pressure ulcer grade II

As regards comparison of presence of comorbidities between EBV and CMV seropositive and seronegative, Table 2 shows that patients in seropositive group for EBV and CMV who had no comorbidities (70%) were more than those who had comorbidities (30%) with no statistically significant difference, as regards those had comorbidities in seropositive group, the most common comorbidities were diabetes mellitus (50%) and hypertension (50%); also, these comorbidities were with higher percent in seropositive group more than in seronegative group, but without statistically significant difference. The only comorbidities that had statistically significant difference between seropositive group and seronegative group was kidney disease. Number of patients in seropositive group who had no kidney disease (80%) was more than those who had kidney disease (20%).

As regards comparison of symptoms between both groups, Table 3 shows that clinical symptoms such as fever, which its incidence in seropositive group is higher than in seronegative group with no statistically significant difference between both groups for any clinical symptom.

**Table 3:** Comparison of symptoms between COVID-19 patients with and without CMV or EBV coinfection (*n* = 110)

	Either CMV or EBV				*P*
	Negative (*n* = 100)		Positive (*n* = 10)		
	*N*	%	*N*	%	
Fever					
No	3	3.0%	0	0.0%	1.000
Yes	97	97.0%	10	100.0%	
Cough					
No	11	11.0%	3	30.0%	.115
Yes	89	89.0%	7	70.0%	
Dyspnea					
No	70	70.0%	6	60.0%	.495
Yes	30	30.0%	4	40.0%	
Shortness of breath					
No	91	91.0%	9	90.0%	1.000
Yes	9	9.0%	1	10.0%	
Chest pain					
No	99	99.0%	10	100.0%	1.000
Yes	1	1.0%	0	0.0%	
Headache					
No	96	96.0%	10	100.0%	1.000
Yes	4	4.0%	0	0.0%	
Bony ache					
No	78	78.0%	7	70.0%	.692
Yes	22	22.0%	3	30.0%	
Malaise					
No	96	96.0%	10	100.0%	1.000
Yes	4	4.0%	0	0.0%	

As regards laboratory parameters in the comparison between both groups, Table 4 shows that blood picture parameters lower values in seropositive group than in seronegative group without statistically significant difference except for platelets (*P* value = 0.021). Also, there were higher values for CRP, serum ferritin, D-dimer, ESR, ALT, AST, and PT in seropositive group than in seronegative group, but with no statistically significant difference between both groups. But there were lower serum albumin values in seropositive group than in seronegative group with statistically significant difference (*P* value = 0.039).

**Table 4:** Comparison of laboratory investigations results between patients with and without CMV or EBV coinfection (*n* = 110)

	Either CMV or EBV				*P*
	Negative		Positive		
	Mean ± SD (min–max)	Median (IQR)	Mean ± SD (min–max)	Median (IQR)	
TLC	9.3376 ± 9.3454 (2.6–89.8)	8 (4.9–11.1)	6.38 ± 3.0195 (0.7–10.6)	6.3 (4.8–9.3)	.257
Lymphocytes	1.4588 ± 1.0757 (0.34–6.8)	1.1 (0.8–1.6)	1.1333 ± 0.9802 (0.04–3.17)	0.94 (0.8–1)	.271
Neutrophils	7.2056 ± 8.8012 (0–82.8)	5.505 (3.7–8.8)	4.4422 ± 2.5959 (0.58–7.7)	4.6 (2.1–5.91)	.200
RBCs	4.8831 ± 0.6437 (3.23–6.44)	4.84 (4.4–5.24)	4.8771 ± 1.0164 (2.97–6.29)	4.83 (4.67–5.59)	.781
Hb	12.8786 ± 1.803 (7.9–18.5)	13 (12–14.2)	12.26 ± 2.9923 (6.9–15.5)	13.45 (10–13.9)	.992
Platelets	205 ± 88 (41–439)	186 (141–257)	139 ± 49 (51–199)	141 (117–185)	.021*
CRP	79.0638 ± 60.4708 (2.3–384)	96 (32–96)	125.81 ± 74.96(42.2–241)	96 (70.55–195.1)	.096
S. ferritin	438 ± 359 (10–1200)	340 (109–752)	1407 ± 1617 (245–4805)	621 (395–1776)	.053
D-dimer	1124 ± 1333 (187–7237)	636 (440–1126)	1076 ± 586 (450–1970)	916 (598–1579)	.223
1st_h_ESR	23 ± 5 (15–26)	25 (20–26)	90 ± 17 (80–110)	80 (80–110)	.057
2nd_h_ESR	35 ± (35–35)	35 (35–35)	106 ± (106–106)	106 (106–106)	1.000
Total bilirubin	1.0327 ± 0.6929 (0.4–3.8)	0.8 (0.6–1.15)	1 ± 0.6693 (0.5–2.3)	0.85 (0.5–1)	.919
Direct bilirubin	0.5922 ± 0.5879 (0.1–2.8)	0.3 (0.3–0.7)	0.3833 ± 0.2787 (0.1–0.8)	0.35 (0.1–0.6)	.538
ALT	74 ± 59 (14–326)	58 (36–81)	83 ± 94 (14–338)	50 (42–94)	.860
AST	79 ± 42 (29–315)	66 (53–96)	118 ± 201 (13–684)	53 (40–92)	.128
Serum albumin	3.815 ± 0.5713 (2.2–4.9)	3.8 (3.5–4.2)	3.1333 ± 1.0113 (1.8–4.8)	3.05 (2.6–3.5)	.039*
ALP	87 ± 36 (33–182)	76 (72–97)	106 ± 32 (62–150)	103 (86–134)	.177
GGT	118 ± 139 (23–532)	60 (41–148)	105 ± 109 (28–182)	105 (28–182)	.933
PT	15.1785 ± 1.8999 (12.3–21.4)	15.4 (13.3–16.1)	16 ± 1.186 (14.5–17)	16.25 (15.05–16.95)	.258
INR	1.251 ± 0.2372 (0.85–1.95)	1.27 (1.06–1.36)	1.252 ± 0.1297 (1–1.42)	1.27 (1.2–1.37)	.810

Mann-Whitney* U* test was used

*TLC* total leucocytic count, *RBCs* red blood cells, *Hb* hemoglobin, *CRP* C-reactive protein, *ESR* erythrocyte sedimentation rate. *ALT* alanine aminotransferase, *AST* aspartate aminotransferase, *ALP* alkaline phosphatase, *GGT* gamma glutamyl transferase, *PT* prothrombin time, *INR* International Normalized Ratio

The median of hospital stay in seropositive group was (15 days) nearly double that of seronegative group with statistically significant difference between both groups.

As regards drugs intake in the comparison between both groups, Table 5 shows that steroids were taken in seropositive group in higher percentage than in seronegative group with no statistically significant difference. On the other hand, vitamins and antibiotics were taken in seronegative group than in seropositive group with statistically significant difference.

**Table 5:** Drugs intake among COVID-19 patients with and without CMV or EBV coinfection (*n* = 110)

	Either CMV or EBV		*P*
	Negative	Positive	
	*N* (%)	*N* (%)	
Steroids			
Not taken	36 (36)	3 (30)	1.000
Taken	64 (64)	7 (70)	
Anti-inflammatory drugs			
Not taken	11 (11.2)	3 (33.3)	.094
Taken	87 (88.8)	6 (66.7)	
Anti-viral drugs			
Not taken	11 (11)	1 (10)	1.000
Taken	89 (89)	9 (90)	
Anti-coagulant drugs			
Not taken	13 (13)	2 (20)	.624
Taken	87 (87)	8 (80)	
Vitamins			
Not taken	10 (10)	4 (40)	.023*
Taken	90 (90)	6 (60)	
Antipyretic and analgesics			
Not taken	9 (9.2)	3 (33.3)	.062
Taken	89 (90.8)	6 (66.7)	
Antibiotic			
Not taken	3 (3)	3 (30)	.010*
Taken	97 (97)	7 (70)	

Fisher’s exact test was used; (*) *P* value (≤ 0.05) is considered statistically significant

In EBV-VCA IgM and CMV IgM seropositive group, 4 (40.0%) are moderate COVID-19 cases, 4 (40.0%) severe cases, 2 (20.0%) critical cases on admission. Seven (70.0%) of them improved and discharged, 3 (30.0%) need ICU admission and died, as illustrated in Tables 6 and 7 respectively.

**Table 6:** Severity of COVID-19 disease among patients with and without CMV or EBV coinfection (*n* = 110)

	Either CMV or EBV				*P*
	Negative (*n* = 100)		Positive (*n* = 10)		
	*N*	%	*N*	%	
Severity of COVID-19 disease					
Mild	5	5.0%	0	0.0%	.471
Moderate	52	52.0%	4	40.0%	
Severe	35	35.0%	4	40.0%	
Critical	8	8.0%	2	20.0%	

Fisher’s exact test was used

**Table 7:** Comparison between COVID-19 patients with and without CMV or EBV coinfection as regards admission and discharge data (*n* = 110)

	Either CMV or EBV				*P*
	Negative (*n* = 100)		Positive (*n* = 10)		
	*N*	%	*N*	%	
Outpatient clinic					
No	93	93.0%	9	90.0%	.546
Yes	7	7.0%	1	10.0%	
Inpatient improved and discharged					
No	11	11.0%	3	30.0%	.115
Yes	89	89.0%	7	70.0%	
Need ICU admission and died					
No	89	89.0%	7	70.0%	.115
Yes	11	11.0%	3	30.0%	

Fisher’s exact test was used

## Discussion

EBV is latent in 90% of people approximately, and this is the highest rate among herpes viruses [14]. In individuals with severe COVID-19 illness, reactivation of viruses, such as herpes simplex, EBV, and CMV, occurs. The cause which has been suggested for this reactivation is the functional exhaustion of cytotoxic lymphocytes [15, 16]. COVID-19 can produce cellular immune dysfunction [16]; therefore, it can stimulate reactivation of latent viruses. Several studies have demonstrated a high incidence of EBV reactivation in COVID-19 patients [17–19].

Our study found that 10 (9%) COVID-19 patients were EBV-VCA IgM and CMV IgM seropositive, 5 (4.5%) COVID-19 patients were EBV-VCA IgM seropositive, and 5 (4.5%) COVID-19 patients were CMV IgM seropositive.

While the results in the study of Xie et al. (2021) which found that 17 (13.3%) were diagnosed with EBV infection in 128 critically ill COVID-19 patients [20]. Probably, the difference in the results because they established their study on critically ill COVID-19 patients only while in this study, the sample include all spectrum of illness from mild to critical COVID-19 cases.

Also, Im et al. (2022) reported that EBV viremia was found in 16.7% of 269 COVID-19 patients [19]. The difference in the results may be because their sample was larger than our sample, and their results depend on detection of EBV viremia, using the Real-Q EBV Quantification Kit (BioSewoom, Inc., Seoul, Republic of Korea) with 72 copies/mL cut-off value for EBV viremia while we use EBV-VCA IgM in the detection of EBV infection.

In addition, Gold et al. (2021) found that EBV reactivation was in 66.7% (20/30) of long-term long COVID patients versus 10% (2/20) of long-term control patients, based on positive titers for EBV VCA IgM or EBV EA-D IgG [21]. The difference in results may be because they used EBV VCA IgM or EBV EA-D IgG to detect EBV infection reactivation, and they found 18 of the long-term long COVID-19 patients were positive for EBV EA-D IgG, 1 of which was also EBV VCA IgM-positive. Two additional long-term long COVID-19 patients were positive for EBV VCA IgM but not EBV EA-D IgG, and the two patients positive for EBV reactivation in the long-term control group were positive for EBV EA-D IgG only, they caught more cases of EBV reactivation by EBV EA-D IgG, while we only used EBV VCA IgM in the detection of EBV infection.

On the other hand, Chen et al.’s (2021) study disagreed with our results as they found negative CMV IgM antibody in COVID-19 patients in their study [22], probably because their study included relatively small number (67 COVID-19 patients) than our sample (110 COVID-19 patients).

Also, Paolucci et al.’s (2021) study is in disagreement with our results about CMV IgM as they reported that CMV reactivation was never observed, they do CMV PCR [18] but in this study, it was not done. This difference in these results could be attributed to the variability in COVID-19 infection nature and the sociodemographic difference of patients.

In the current study, regarding the symptoms, the incidence of fever in the EBV and HCMV seropositive group was apparently higher than that in the EBV and HCMV seronegative group. Also, no statistically significant difference for other symptoms between both groups. This was in agreement with Naendrup et al.’s (2021) study which reported persisting fever in COVID-19 patients with EBV and CMV reactivation [11]. Also, these findings agreed with the findings in Chen et al. (2021), beside that they found statistically significant difference for fever between both groups [22].

As regards laboratory parameters in the current study, the platelets, albumin, serum ferritin, D-dimer, and C-reactive protein show higher values in seropositive group than in seronegative group but not statistically significant. Thrombocytopenia conditions and immune thrombocytopenia reported with EBV and CMV infection in many studies such as Wu et al. (2013) and Zhang et al. (2021) which agree with our findings in the current study [23, 24].

This is in disagreement with Chen et al.’s (2021) study which reported that blood routine examination and blood biochemistry results reveals no significant differences between EBV/SARS-CoV-2 coinfection and SARS-CoV-2 infection alone patients [22]. Also, this was in disagreement with Naendrup et al.’s (2021) study which reported that there were no correlations between EBV and CMV reactivations and neutrophil-to-lymphocyte ratio [11].

This finding is in agreement with Xie et al.’s (2021) study which reported that patients with EBV reactivation had lower albumin and higher incidence of hypoproteinaemia in EBV group than in non-EBV group [20].

These findings were not agreed with those in Chen et al.’s (2021) study which reported that no significant difference in liver function tests between EBV/SARS-CoV-2 coinfection patients and SARS-CoV-2 infection alone patients except for aspartate aminotransferase (AST) which had significantly higher results in EBV/SARS-CoV-2 coinfection patients than that in SARS-CoV-2 infection alone patients [22].

On the other hand, Naendrup et al.’s (2021) study reported that there was no correlation between EBV and CMV reactivations and the transaminases as well as bilirubin were detected [11].

These higher values of C-reactive protein also shown in EBV/SARS-CoV-2 coinfection patients in Chen et al.’s (2021) study and were higher than that of SARS-CoV-2 infection alone patients [22]. Higher D-dimer and CRP in EBV seropositive COVID-19 patients than in EBV seronegative patients also reported in Xie et al. (2021) [20].

On the other hand, this was in disagreement with Naendrup et al.’s (2021) study which reported that there was no correlation between EBV and CMV reactivations and C-reactive protein, and ferritin levels [11].

In the current study, seropositive group had received higher doses of steroids than seronegative group. This was in agreement with Naendrup et al.’s (2021) study which stated that the use of high‐dose corticosteroids as a risk factor for herpes virus reactivation [11].

Also, these findings agreed with Chen et al.’s (2021) study which reported that the EBV/SARS-CoV-2 coinfection individuals were more likely to be received corticosteroid therapy by doctors than the SARS-CoV-2 infection alone patients [22].

In addition, Im et al.’s (2022) study reported that steroid administration is often prolonged in patients with severe COVID-19. In addition, host immunity may be compromised because of critical illness. EBV viremia can persist at high levels in these cases [19].

Moreover, in the current study, as regards period of admission in the comparison between EBV and CMV seropositive and seronegative groups, the median of hospital stay for the seropositive group was (15.0) double that for the seronegative group (7.0) with statistically significant difference (*P* value = 0.019). These data were in agreement with Chen et al. (2021) but without statistically significant difference. Also, agree with Simonnet et al.’s (2021) study which that EBV reactivation in critically ill COVID-19 patients was associated with a longer duration of intensive care unit stay [25].

The current study did not show increased disease severity in individuals with coinfection of EBV, CMV, and SARS‐CoV‐2 and the results demonstrated no differences in disease outcome, may be due to the small sample. These results are in agreement with Blumenthal et al. (2021), who found that individuals with coinfection of EBV and SARS‐CoV‐2 did not show increasing in disease severity and the results showed no differences in terms of viral load and disease outcome [26]. Larger scale of studies including COVID-19 patients with either normal or elevated liver enzymes values could give more data about the effect of CMV and EBV coinfection on the incidence of elevation of liver enzymes in such patients.

## Conclusion

Coinfection of EBV and CMV has been found in Egyptian COVID-19 patients. COVID-19 disease severity and clinical outcome were not affected by EBV and CMV coinfection, but those patients had higher hospital stay duration.

### Study limitations

The limited number of studied patients and the dependence on serological tests in CMV and EBV diagnosis rather confirmation by Rt-PCR.

## Data Availability

All data generated or analyzed during this study are included in this published article.

## Abbreviations

EBV: Epstein–barr virus
CMV: Cytomegalovirus
HRCT: High-resolution computed tomography
HCMV: Human cytomegalovirus
VCA: Viral capsid antigen
ELISA: Enzyme-linked immunosorbent assay
COVID-19: Coronavirus disease 2019
SARS‐CoV‐2: Severe acute respiratory syndrome -coronavirus-2
DM: Diabetes mellitus
HTN: Hypertension
IHD: Ischemic heart disease
BA: Bronchial asthma
COPD: Chronic obstructive pulmonary disease
HF: Heart failure
CBC: Complete blood count
CRP: C-reactive protein
ESR: Erythrocyte sedimentation rate
ALT: Alanine aminotransferase
AST: Aspartate aminotransferase
ALP: Alkaline phosphatase
GGT: Gamma glutamyl transferase
PT: Prothrombin time
INR: The international normalized ratio
RT-PCR: Real-time reverse transcription polymerase chain reaction
EBV EA: Epstein–Barr virus early antigen
